# Phenotyping Tumor Heterogeneity through Proteogenomics: Study Models and Challenges

**DOI:** 10.3390/ijms25168830

**Published:** 2024-08-14

**Authors:** Diletta Piana, Federica Iavarone, Elisa De Paolis, Gennaro Daniele, Federico Parisella, Angelo Minucci, Viviana Greco, Andrea Urbani

**Affiliations:** 1 Department of Basic Biotechnological Sciences, Intensivological and Perioperative Clinics, Università Cattolica del Sacro Cuore, 00168 Rome, Italy; diletta.piana@unicatt.it (D.P.); federica.iavarone@unicatt.it (F.I.); federico.parisella01@icatt.it (F.P.); 2 Departmen Unity of Chemistry, Biochemistry and Clinical Molecular Biology, Department of Diagnostic and Laboratory Medicine, Fondazione Policlinico Universitario A. Gemelli IRCCS, 00168 Rome, Italy; elisa.depaolis@policlinicogemelli.it (E.D.P.); angelo.minucci@policlinicogemelli.it (A.M.); 3 Departmental Unit of Molecular and Genomic Diagnostics, Genomics Core Facility, Gemelli Science and Technology Park (G-STeP), Fondazione Policlinico Universitario A. Gemelli IRCCS, 00168 Rome, Italy; 4 Phase 1 Unit, Fondazione Policlinico Universitario A. Gemelli IRCCS, 00168 Rome, Italy; gennaro.daniele@policlinicogemelli.it

**Keywords:** cancer, personalized medicine, proteomics, proteogenomics

## Abstract

Tumor heterogeneity refers to the diversity observed among tumor cells: both between different tumors (inter-tumor heterogeneity) and within a single tumor (intra-tumor heterogeneity). These cells can display distinct morphological and phenotypic characteristics, including variations in cellular morphology, metastatic potential and variability treatment responses among patients. Therefore, a comprehensive understanding of such heterogeneity is necessary for deciphering tumor-specific mechanisms that may be diagnostically and therapeutically valuable. Innovative and multidisciplinary approaches are needed to understand this complex feature. In this context, proteogenomics has been emerging as a significant resource for integrating omics fields such as genomics and proteomics. By combining data obtained from both Next-Generation Sequencing (NGS) technologies and mass spectrometry (MS) analyses, proteogenomics aims to provide a comprehensive view of tumor heterogeneity. This approach reveals molecular alterations and phenotypic features related to tumor subtypes, potentially identifying therapeutic biomarkers. Many achievements have been made; however, despite continuous advances in proteogenomics-based methodologies, several challenges remain: in particular the limitations in sensitivity and specificity and the lack of optimal study models. This review highlights the impact of proteogenomics on characterizing tumor phenotypes, focusing on the critical challenges and current limitations of its use in different clinical and preclinical models for tumor phenotypic characterization.

## 1. Introduction

Tumor heterogeneity (TH) is a general term that refers to the differences in the molecular and phenotypic characteristics of tumoral cells within the same tumor or among different tumors, which are often related to its aggressive nature. It includes molecular, cellular, and architectural variability [1]. According to the National Cancer Institute’s dictionary, a tumor is an abnormal mass of tissue that forms when cells grow and divide more than they should or do not die when they should [2]. The tissue mass is primarily characterized by intra-tumor heterogeneity (ITH) from the outset, which is also the by-product of tumor progression [3]. However, the development of different tumor cell clones characterized by different architecture, metastatic properties, and drug resistance is regulated not only by the accumulation of somatic genetic mutations (also defined tumoral mutational burden—TMB) [4,5] but also by a portrait of epigenetic and phenotypic changes due to the intra- and peritumorous tumor microenvironment (TME) and immunopharmacological therapy [6,7].

All facets of ITH, including the genetics and protein features and TME, result in various patient responses to therapy and possible relapses. Therefore, different tumor phenotypes are crucial for treatment response [8,9,10], and gaining a full understanding of tumoral heterogeneity and its phenotypic features may significantly impact both diagnosis and therapy. Single genomic analyses by Next-Generation Sequencing (NGS) technologies have made it possible to profile a large number of cancer-related mutations [11,12,13].

However, understanding the actual expression of TMB and the effects of certain mutations remains challenging due to the difficulties in translating genomic information into protein-level functions [14], which are crucial for determining phenotype. As Aebersold’s studies show, the variants of HeLa cell lines exhibit significant biological variability, highlighting the challenge in understanding genotype–phenotype correlations in cancer cells [15]. Genetic variability has an intricate and non-linear impact on transcription profiles, proteome and protein turnover [15]. Moreover, protein dynamicity, including isoforms and Post-Translational Modifications (PTMs), increases the complexity to the proteome, accurately reflecting the essential changes in tumor phenotypes [16,17]. These variations cannot be fully explained through genomic and transcriptomic sequencing analyses [18]. Advances in proteomics, particularly with high-resolution mass spectrometry (HRMS), have led to a new molecular integrative approach called proteogenomics, which has been instrumental in characterizing the molecular complexity of tumor phenotypes in greater depth [19].

This innovative approach has been rapidly developing in cancer research, highlighting a new horizon in precision oncology, where a better understanding of tumor phenotypes can lead to better patient stratification and specific target therapies. Proteogenomics is optimal in this regard, as it allows the investigation of tumor heterogeneity in a more advanced manner by integrating proteomics with genomic and transcriptomic data obtained from NGS technologies and HRMS to define functional correlations between genes and proteins [20,21].

However, the complete knowledge of heterogeneous tumor phenotypes has often been hampered by the sensitivity of the molecular analyses used and the lack of an optimal model to represent a highly dynamic ITH.

Thus, given the complex nature of tumor heterogeneity and certain limitations of the molecular technologies used in proteogenomic analyses, overcoming these issues could lead to better phenotyping of tumor heterogeneity.

This review focuses on the value of proteogenomic approaches in characterizing the complex molecular differences of various tumor phenotypes, primarily considering the literature from the past five years. In particular, critical challenges and current limitations of its use will be discussed, focusing on different clinical and preclinical models used for tumor phenotypic characterization.

## 2. Proteogenomics

Proteogenomics is an innovative and evolving approach which integrates data from genomics and transcriptomics, such as DNA mutations, epigenetic regulation, and RNA expression along with proteomic data including proteins, their expressions, and PTMs [22] to gain a comprehensive understanding of complex biological phenotypes like tumors [21,23]. Large-scale genomic studies and new NGS technologies are instrumental in identifying the origin of cancers and suggesting “driver genes” within analyzed tumors [12,13]. This aspect has revolutionized cancer drugs development. Patients with specific molecular profiles are included in clinical trials based on their tumor signature (umbrella trials) or without such reliance (basket trials), depending on the likelihood that genomic biomarkers will predict response to targeted therapies. Examples of successful targeted therapeutics include those for *BRAF*-mutated melanoma [24] and *NTRK*-altered (neurotrophic receptor tyrosine kinase) tumors [25].

However, the outcomes of targeted therapies are not always predicted by the mere presence of a specific mutation [26]. Resistance mutations and tumor heterogeneity indicate that gene sequence analyses alone are insufficient [18]. Different phenotypes are often revealed through the study of proteins, PTMs, and proteoforms [16,17]. Therefore, understanding the expression, function, and interactions of proteins is crucial for elucidating the molecular mechanisms biological processes.

Affinity ligands-based proteomic technologies, such as Reverse Phase Protein Array (RPPA), Protein Expression Array (PEA) and SOMAscan (slow off-rate modified aptamers), have been developed to identify and target several proteins, facilitating the detection of potential therapeutic targets [27,28,29]. However, these techniques show some limitations, particularly in detecting PTMs due to variability in the affinity versus avidity of antibodies or aptamers used in the assays.

Conversely, MS-based proteomic analysis, which can detect the dynamic and complex proteome—including its PTMs—enables a more detailed study of tumor phenotypes. This includes the investigation of phosphoproteomes, glycoproteomes, acetylproteomes, and ubiquitinomes, which are fundamental for understanding various biological processes related to tumor survival, death, and signaling [16].

**Figure 1:**
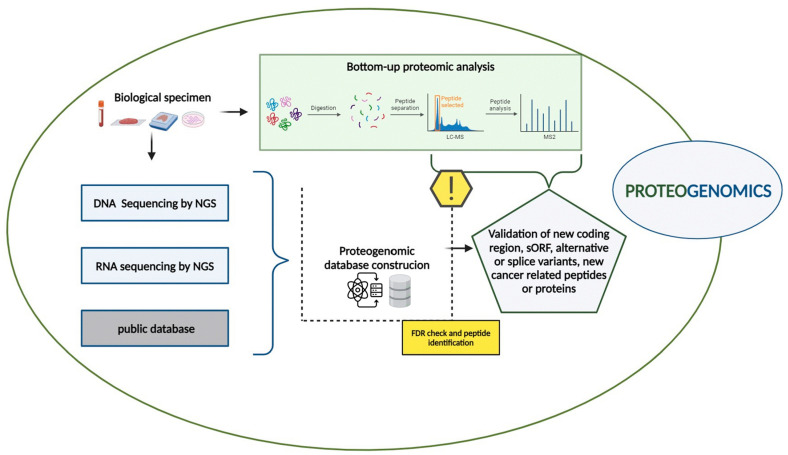
Proteogenomic workflow. The proteogenomic experimental workflow, combining genomic, transcriptomic and proteomic data, is described (created with BioRender.com).

In this context, proteogenomic approaches have emerged as a crucial bridge between both genetic and phenotypic variability, allowing the deciphering of biological mechanisms in cancers, leading to the identification of clinically applicable biomarkers and new therapeutic targets [21,23]. The proteogenomic workflow integrates genomic, transcriptomic, and proteomic data obtained from the same biological samples such as tissue biopsies, liquid biopsies, and cell cultures, as described in Figure 1.

Whole Genome Sequencing (WGS), Whole Exome Sequencing (WES), and RNA sequencing by NGS are primarily to identify genomic and somatic mutations, including single nucleotide variant (SNV), insertion and deletions (Indels), Copy Number Alterations (CNAs), and transcriptomic data [30]. Additionally, public databases such as NCBI Reference Sequence (RefSeq) and Ensembl [31,32] provide valuable resources for gathering genomic and transcriptomic information.

Simultaneously, bottom–up proteomic analyses are performed on the same samples using liquid chromatography-tandem mass spectrometry (LC-MS/MS). In this process, proteins are first isolated from biological samples and then digested into peptides, typically using trypsin or other enzymes. The resulting peptide mixture undergoes LC-MS/MS analysis, and the related MS and MS/MS spectra are collected and analyzed with curated databases.

A critical step in proteogenomics is the creation of a custom proteomic database that integrates genomic and transcriptomic data. Nucleotide sequences obtained from public repositories or sample sequencing, such as DNA, RNA, or Ribo-sequencing, are translated into amino acid sequences to build this database [33]. Several translation methods such as six-frame translation for DNA sequences and three-frame translation methods for RNA sequences are employed [34].

This custom proteomic database significantly enhances protein identification by facilitating the discovery of proteins not represented in standard reference databases. These include unannotated proteins resulting from alternative splicing, splice variants, and neoantigens derived from Splicing Variants (SVs) or encoded by alternative Open-Reading Frames (ORFs) [20].

### 2.1. From Single-Cell Analyses to Proteogenomics

Single-cell analysis has become crucial in medical and biological research, opening up new perspectives in cell biology and medicine, particularly in cancer research [35]. It has significantly contributed to the more precise identification of tumor heterogeneity [36].

For example, a single-cell RNA sequencing (scRNA-Seq) study of high-grade serous tubal ovarian cancer revealed different cellular phenotypes associated with poorer prognostic outcomes. This underscores the importance of distinguishing stromal components from the bulk tumor for the classification of molecular subtypes [37]. Similarly, Xu’s scRNA-seq study of high-grade serous ovarian cancer demonstrated significant differences in the immune infiltrate composition, such as the presence and activation of tumor-associated macrophages (TAMs) and T-cells, providing valuable information for defining optimal therapeutic strategies [38]. Notably, the differential expressions of thirty-eight genes in epithelial-to-mesenchymal transition (EMT) between normal and tumoral cells has been revealed [38].

Although scRNA-Seq is currently being used in clinical trials to evaluate the safety of novel drugs in tumors, as reviewed by Lei et al. in [35], several limitations need to be considered.

While scRNA-seq provides valuable information regarding tumor cellular heterogeneity at the transcriptomic level, it cannot describe the epigenetic and proteomic status of cells.

The lack of integration with epigenetic or methylome techniques impacts the applications of scRNA-seq in detecting epigenetic regulations such as histone modifications and DNA methylation, as well as long non-coding RNAs [39], which are increasingly recognized as relevant in this context [40].

Furthermore, mRNA transcripts levels are partially correlated with the protein abundances [41,42]. The relationship between mRNA and protein levels is complex, and it is influenced by various mechanisms such as post-transcriptional and post-translational modification as well as the interaction between proteins [43,44,45]. Integrating “omics” data, including epigenomic and proteomic data, is essential for obtaining a comprehensive review of biological mechanisms and specific cellular phenotypes [46].

While single-cells genomics and transcriptomics technologies initially led advances in the field, single-cell proteomics (SCPs) is now developing in the wake of scRNA-Seq to identify and quantify proteins at cell levels [47].

Over the last few years, different SCP technologies have emerged such as Single-Cell Barcode Chip (SCBC), single-cell Western blotting (scWB), Cytometry by Time-Of-Flight (CyTOF), and Cellular Indexing of Transcriptomes and Epitopes by sequencing (CITE-Seq) and single-cell proteomics by mass spectrometry (SCP-MS) as reviewed by Xie and Ding in [48]. Most SCP applications are targeted proteomic analyses, where target proteins are detected by antibodies, such as in CyTOF, or by aptamers or oligonucleotide-labeled antibodies in CITE-seq and RNA Expression And Protein Sequencing (REAP-Seq) analyses [48,49,50]. Unlike CyTOF, CITE-seq and REAP-seq allow the simultaneous measurement of the transcriptome at a single-cell level but are restricted to the detection of surface proteins only (Table 1).

Advancements in SCP-MS techniques have expanded the possibilities for untargeted proteomic analysis at the single-cell level, allowing a broader exploration of the proteome without being confined to specific protein targets.

SCP-MS technologies, such as nanodroplet Processing in One pot for Trace Samples (nanoPOTS), for sample processing, and single-cell proteomics by MS (ScoPE MS) and its evolution, ScoPE MS2, have raised interest in the development of the field of single-cell ProteoGenomics (scPG) [51]. This approach could be helpful for deciphering ITH within tumoral cells. Compared to bulk analyses on clinical models such as tissue biopsies, scPG could provide a more comprehensive view of individual cells, improving the characterization of molecular changes during physiological and pathological processes [51].

For an overview of the advantages, limitations, and troubleshooting strategies of these techniques, refer to Table 1.

**Table 1:** Advantages, limitations, and troubleshooting of single-cell techniques.

Techniques	Advantages	Limitations and Troubleshooting	References
ScRNA-seq	Profile the transcriptomes of individual cells, multiplexed analyses, high throughput	RNA amplification bias, cell capture processes, detecting of non-coding RNA; improve the process and methods for controlling batch effects	[35,52]
SCBC	Rapid, multiplexed analysis up to 42 proteins, detection of secreted and intracellular proteins	Limited by antibody availability, high cost; improve sample preparation techniques, regular updates and validation of encoded antibody libraries	[48,53]
scWB	High-resolution profiling cell of surface and cytoplasmatic proteins at the single-cell level, rapid	Low throughput, detection of low abundance proteins and small molecular weight proteins, antibody specificity; use high-quality antibodies, improve the antibody incubation performance	[48,53]
CyTOF	Measures multiple parameters with high sensitivity	High-quality antibodies, is expensive, not optimal for living cells, involves complex data analysis; validate antibody quality, optimize experimental conditions, unsupervised and bioinformatic approaches for data analysis	[48,49,53,54]
CITE-Seq and REAP-seq	Combines proteomic and transcriptomic profiling at single-cell level, detect 3′ RNA ends	Limited by antibody availability, limited to detection of surface proteins, complexity of data analysis; employ high-quality antibodies for detection of intracellular protein, use advanced bioinformatics tools	[48,50]
SCP-MS	Extensive proteome coverage through multiplexing	Isolation, digestion, protein transfer processes; improve sample preparation, enhance ion accumulation techniques	[48,51,53,55,56]

### 2.2. Clinical and Preclinical Study Models to Study Tumor Heterogeneity

#### 2.2.1. Tissue from Biopsies and Resection Specimens

Fresh Frozen Tissue (FFT), Optimal Cutting Temperature (OCT), and Formalin-Fixed Paraffin-Embedded tissue (FFPE) are the most widely used tissue biopsy samples for clinical molecular analysis [57] and have been considered the “gold standard” for cancer diagnosis and research for decades [58].

However, FFPE, FFT and OCT provide a static image of the analyzed tumor. These techniques make it challenging to monitor molecular changes over time, such as the dynamic pattern of mutations occurring during clonal evolution or the process of metastatization and the dynamism of protein expression [58,59].

The Variant Allele Frequency (VAF) plays a valuable role in this context. It could be used to speculate about dynamic changes between primary tumors and their relapses. VAF is useful for evaluating the origin of mutations, and in the evaluation of mutation origin in terms of germline or somatic origin, especially when a normal reference sample is not available [60].

In this context, proteogenomics emerges as a promising approach, allowing for a comprehensive understanding of the molecular alterations driving tumorigenesis and the disease progression of several tumors, such as breast cancer [61].

However, the data generated by proteogenomic analyses using these models often provide an incomplete understanding of the genomic/transcriptomic and proteomic heterogeneity of cells as they typically reflect an average of the molecular characteristics across a cell population [62,63].

To address this issue, multiregional sampling and single-cell analyses have been employed in several cancer studies to gain a better understanding of ITH and to reconstruct the cancer evolutionary history in different malignancies, revealing the clonal heterogeneity of different cell types [10,64].

Nevertheless, pre-analytical conditions also pose limitations. The transition of tumor tissue samples from surgical resection to the molecular laboratory, along with the modifications that tissue specimens undergo before analysis, can affect the results [65].

For instance, isolating and analyzing single cells from different sections and time points of tumor biopsies can lead to cell loss or biases in gene expression [66]. In addition, the interference of the OCT polymer can suppress ionization during MS/MS analyses, resulting in reduced peptide identification [67]. FFPE samples can undergo modifications, such as crosslinking due to formaldehyde, which can alter the DNA structure and protein yield, potentially masking accessible sites for trypsin digestion [68,69].

Despite these challenges, improvements have been made in protocols for proteome analysis on FFPE tissues, leading to a more efficient decrosslinking of proteins and increased protein yield for digestion [57]. As a result, while tissue biopsies remain the most common clinical model for tumor diagnosis, they still have limitations in accurately characterizing the complex molecular dynamics of tumor phenotypes. This highlights the need for less invasive models that can better capture the molecular dynamics of tumor heterogeneity.

#### 2.2.2. Liquid Biopsies

In the last few years, liquid biopsies (LBs) have been proposed as a new approach that overcomes several challenges related to solid biopsy analyses. LBs consist of the evaluation of biological fluids with the final aim of analyzing tumor molecular profiles and real-time changes, thanks to longitudinal sampling. Compared to solid tumor biopsies, an LB ideally represents a simple and minimally invasive strategy [70,71]. The most common sample type used in LB is blood, even if many other biological fluids can be used for molecular biomarkers characterization, such as urine [72,73], cerebrospinal fluid [74,75,76], pleural [77,78], and ascites [79] effusions. LB encompasses different molecular biomarkers like circulating cell-free DNA (cfDNA), circulating tumor DNA (ctDNA), circulating cell-free RNA (cfRNA), extracellular vesicles (EVs), proteins, circulating microRNA (miRNA), and circulating tumor cells (CTCs). These biomarkers are broadly released into the blood or in other biofluids by tumor cells [80]. In clinical settings, cfDNA and ctDNA, which represent the tumoral fraction of cfDNA, find wider application [81], while the use of the other biomarkers still needs further implementations.

From a methodological point of view, LB approaches for genomic analyses can be designed in tumor-driven or in tumor-agnostic manners. In fact, an LB can be used to monitor cancer genomics evolution after a prior genotyping of tissue samples (e.g., by using a targeted single-gene approach) or without a previous tumor analysis (e.g., with a more comprehensive multigene panel) [82]. The main genomics methodologies available for these purposes are the droplet digital polymerase chain reaction (ddPCR), Beads Emulsion Amplification and Magnetics (BEAMing), Tagged-Amplicon deep Sequencing (TAm-Seq), CAncer Personalized Profiling by deep Sequencing (CAPP-Seq), Whole Genome Bisulfite Sequencing (WGBS-Seq), Whole Exome Sequencing (WES), and Whole Genome Sequencing (WGS) [83]. Both ddPCR and BEAMing are PCR-based detection techniques that analyze specific target mutations with high specificity and sensitivity, making them ideal for detecting few known molecular alterations [83,84,85] (Table 2). In contrast, NGS-based CAPP-Seq and TAm-Seq offer broader mutation profiling that is suitable for monitoring tumor evolution and resistance mutations [86,87].

However, the low concentration of ctDNA and the variability in ctDNA release can introduce false positive results and reduce the sensitivity of CAPP-seq [86]. Additionally, the effectiveness of TAm-Seq relies on pre-characterizing the genomic regions of interest [83]. This requirement limits its use to cases where specific mutations are already known, reducing its flexibility for novel mutation discovery. WES and WGS provide comprehensive genomic information and are mostly used in proteogenomics approaches, which is essential for discovering novel mutations and understanding the full genomic landscape of tumors [83].

At present, LBs have emerged as a groundbreaking tool mainly in the early diagnosis of low-shedding cancer and in the subsequent monitoring of Minimal Residual Disease (MRD) and acquired resistance mutations, especially in cases of tissue biopsies unavailability [90,91]. In the context of clonal evolution and tumor heterogeneity monitoring, LBs are capable of characterizing the molecular and phenotypic dynamism of the tumor, making them an ideal molecular approach for ITH evaluations. The evolutionary pressure, mainly related to therapy administration, represents the molecular driver of sub-clonal transformation with the coexistence of novel molecular signatures within the same tumoral tissue or in metastasis sites [92]. In this context, solid biopsy can under-represent the overall molecular characteristics of the ITH, limiting the rate of detection of biological and clinically relevant mutations.

Several successful examples of ITH analyses using LBs are available in the literature in different clinical contexts as gastrointestinal [93], non-small cell lung cancer (NSCLC) [94], bladder [95], breast [96,97], colorectal [98], and melanoma [99] tumors. A typical example is the monitoring of acquired resistance in NSCLC using cfDNA after epidermal growth factor receptor tyrosine kinase inhibitors (*EGFR*-TKIs) target therapy, such as the C797S, T790M, L858R, and Del19 *EGFR* variants [100]. Another significant application of cfDNA analysis is the monitoring of breast cancer evolution. In fact, breast cancer is considered one of the tumors with the highest molecular heterogeneity, which is the main cause of resistance to therapies [101]. In Hormone-Receptor-positive (HR+) breast cancer, representing approximately 70% of cases, acquired resistance can be monitored using LB targeted to mutations occurring within the Ligand-Binding Domain (LBD) of the Estrogen Receptor-1 (*ESR1*) gene [102]. Moreover, *ESR1* mutations are associated with inferior Progression-Free Survival (PFS) and Overall Survival (OS) in comparison to non-mutant ESR1 patients treated with exemestane plus everolimus [103].

Beyond ct/cfDNA, CTCs represent one of the first biomarkers analyzed in LB approaches. CTCs are cells shed by the tumor and ideally represent an easy sample type to evaluate. However, their total amount in blood or in other fluids is usually low, requiring advanced isolation methods [83,104]. At present, the only CTC-based LB assay approved by the U.S. Food and Drug Administration (FDA) is the CellSearch, which is a platform that uses EpCAM enrichment in patients with colorectal, breast, or prostate cancer [105]. Jordan et al. reported a successful example of CTCs use in patients with breast cancers. The authors, by monitoring CTCs release, showed a shift from Human Epidermal Growth Receptors 2 (*HER2*) positive to *HER2* negative-CTCs status, which may suggest a clonal shift toward resistance to chemotherapy [106].

In addition, circulating RNA and miRNA were investigated in several studies as non-invasive LB markers. In particular, risk stratification strategies were proposed according to RNA-expression panels in lung [107] and gastric [108] cancers.

Despite the advantages that LB have demonstrated in clinical practice, there are some limitations related to technological and biological issues in the ITH descriptions. The main actual limitations are the potential confounding effect of clonal hematopoietic mutations of indeterminate potential (CHIP) and the lower sensitivity documented for some applications, such as fusion detection at the RNA level [109]. Furthermore, in ITH analyses, it is relevant to keep in mind that tumor-derived biomarkers shedding can differ across tumor sites, affecting the choice of the best sample type and the overall comparison of the experimental and clinical results.

Finally, the alterations at DNA and RNA levels are not enough to reveal modifications in proteins [88], which are the direct performers of most cell functions and the targets of most current cancer therapies. Therefore, deep proteome profiling is more likely to provide valuable and clinically relevant real-time information on cancer progression.

However, proteomic analyses using LB face several challenges.

Blood and plasma are not always optimal biofluid for MS proteomic analysis due to the dynamic concentration range of proteins [57]. To address this issue, protein depletion strategies have been developed to reduce the concentration of high-abundance proteins (Table 2). Furthermore, untargeted proteomic approaches, such as the Data-Independent Acquisition (DIA) and Swath-DIA (sequential window acquisition of all theoretical DIA) approach, have shown promise in improving sensitivity and protein coverage [88]. Despite these advancements, most proteomic studies on liquid biopsies still rely on targeted approaches, such as protein microarrays (RPPAs) or aptamer-based assays (e.g., SOMAscan) [89,110,111].

RPPA was applied in several proteogenomic studies, offering significant advantages such as quantitative measurements of protein expression from small sample volumes and the ability to analyze numerous samples simultaneously [27,88]. However, RPPA is limited by the availability of specific antibodies, which constrains its coverage of the proteome.

On the other hand, SOMAscan utilizes aptamers—short single-stranded DNA or RNA molecules that bind with high affinity to native target proteins [88].

Despite its strengths, SOMAscan also faces challenges, particularly in its inability to cover the entire proteome and capture dynamic protein changes fully.

Consequently, while these targeted proteomic approaches have driven significant advancements in translational research, they still encounter issues related to reproducibility, sensitivity, and accuracy, which limits their ability to provide a comprehensive view of the complex molecular and architectural changes associated with tumor heterogeneity [112].

For a comprehensive summary of the advantages, limitations, and troubleshooting strategies of LB techniques, refer to Table 2.

#### 2.2.3. Organoids

Fresh frozen organoids or 3D cell cultures in vitro are tissue-engineered models that reflect several aspects of the complex structure and function of the corresponding in vivo tissue [113]. They originate from progenitor stem cells or even from a fragment of tissue biopsies. Organoids can spontaneously grow from Adult Stem Cells (ASCs) or derive from Pluripotent Stem Cells (PSCs), which include Embryonal Stem Cells (ESC) or Induced Pluripotent Stem Cells (IPSCs) [114], under specific cell culture conditions. Recently, this model has been used more frequently in cancer research to study tumor heterogeneity due to organoids’ ability to preserve the genetic, proteomic and morphological features of tumors [115,116]. The possibility of creating tumor organoids from tissue specimens, such as Patient-Derived Organoids (PDOs) and Patient-Derived Xenograft (PDX), offers innovative models for studying dynamic multiple dimensional ITH as they reflect the genetic and phenotypic characteristics of the original tumor in patients [117,118]. Since the first organoid tumoral culture was established by Sato et al. in [119], multiple researchers have reported the use of PDOs as a preclinical model to study the heterogeneity of various types of cancers [120,121,122]. This has led to the creation of living biobanks of tumor organoids, which are potentially useful not only for capturing TH but also for predicting drug responses of cancer patients [123], enabling a better screening and stratification of patients for therapies. Among these, a living biobank of advanced colorectal cancer PDO demonstrated a broad range of intrinsic PDO responses to chemotherapy, suggesting that PDO might predict who responds to chemotherapy [124]. A recent study showed that high-grade serous ovarian cancer (HGSOC) PDOs represent a valuable tool for understanding the tumor biology, proposing a possible new ex vivo screening method to identify new drugs to which HGSOC would be vulnerable [125]. Other recent studies on different tumoral phenotypes, such as in liver and lung cancers [126,127], have shown the potential of PDO to predict the molecular, morphological and drug response properties of parental tumors.

However, the generation of PDOs presents significant challenges. Slight differences in responsiveness to therapies could be based on the origin of PDOs, as they are formed from different areas of the sample. Additionally, the technology required to create the PDO model involves some practical difficulties. Unlike PDX models, PDOs lack components of TME, such as fibroblast and Cancer-Associated Fibroblast (CAFs) as well as endothelial and immune cells [118]. TME and immune cells have a key role on heterogeneity and on the success of both chemotherapy and immuno-target therapy. The TME and immune cells play a key role in heterogeneity and in the success of both chemotherapy and immuno-target therapies. Thus, new advances in organoid culture have been made, including microfluidic 3D culture, air–liquid interface culture, and submerged Matrigel culture, in order to capture TME and immune cells [128]. Although several studies have shown improvements in organoid culture with the addition of CAFs and immune cells [129,130], current PDOs remain small due to the lack of vascular elements necessary for nutrient supply [131]. The most innovative clinical application of organoids is the identification of neoepitopes for personalized immunotherapy. Since preclinical models lacked neoantigen-directed therapy [132], a recent multiomic approach by Wang et al. characterized the HLA-class-I neoantigen landscape in hepatobiliary tumors, providing a reliable strategy using tumor organoids to evaluate the immunogenicity of tumor-specific peptides [132]. Another study by Demmers et al. used tumor organoids to analyze the variability in the presentation of HLA class I peptides between different clonal cells from the same colorectal cancer patient, suggesting that a multi-peptide vaccine approach against highly conserved tumor suppressors might be viable in patients with a low mutational burden of cancer [133]. Despite innovative applications in predicting drug responses and in the field of immunotherapy, PDOs have several shortcomings. These include complex protocols, associated costs with the technology, limited data regarding the effect of baseline culture conditions, the addition of extracellular matrix (ECM) and immune cells on growth and response in these heterogeneous organoid culture [134].

Nevertheless, considerable improvements have been made in the field of PDOs to better describe tumor phenotypes. Owing to their intrinsic versatility, ability to model in vivo situations, and rapidly evolving applications, it is expected that organoid technology will have a substantial future impact on basic research and clinical cancer therapy [135].

### 2.3. Applications

Proteogenomics has a broad spectrum of applications. Initially, genome annotation was the primary aim of proteomics and genomics integration studies [136]. Early examples include studies conducted by Yates et al. in 1995 and Choudhary et al. in 2001 [137,138].

In 2004, Jaffe et al. introduced the concept of a “complementary proteogenomic map” for gene annotation [139], where the genome of *Mycoplasma pneumoniae* was translated into a six-frame database, and peptides detected by LC-MS/MS were identified against this custom database. This approach not only improved the validation of known or predicted protein-coding genes but also facilitated the identification of novel open reading frames often overlooked by traditional genomic methods. Over the years, proteogenomic applications have expanded in biomedical research parallel to advancements in NGS, HRMS technologies, advanced bioinformatics tools and repository databases.

In cancer research, The Cancer Genome Atlas (TCGA), the International Cancer Genome Consortium (ICGC), and the Clinical Proteomic Tumor Analysis Consortium (CPTAC) were pioneers of this influential approach [140,141].

The TCGA studies provided significant cancer genomic and transcriptomic classifications through the integration of DNA and RNA sequencing, array-based DNA methylation technologies, and RPPA techniques with multidimensional analyses, as reported by Tomczak et al. in [140]. However, these sequencing-centric studies solely focused on validating known or annotated protein-coding genes, relying on the availability of antibodies, which limited the possibility of capturing the full extent of protein and PTMs expression [142].

With the integration of MS analyses, new proteogenomic studies conducted by CPTAC have shown not only the imbalance in protein–mRNA correlation but also the associations between specific genomic alterations and functional protein changes, identifying PTMs and pathways related to clinical outcomes [18,21,143,144].

To establish these proteogenomic relationships from multiomic data, pathways and correlation analyses are needed. These analyses allow researchers to evaluate the impact of CNAs on mRNA and protein abundance, as well as the interplay between microRNAs and DNA methylation, and clinical data, using machine learning tools and predictive modelling techniques, such as linear and regression models [145].

Recent proteogenomic approaches in lung adenocarcinoma (LUAD) studies by Soltis et al. [146] and non-small cell lung cancer (NSCLC) by Lethio et al. [147] have revealed correlations between RNA, proteins and tumor immune cell composition, providing crucial information for predicting disease progression and therapeutic responsiveness (Table 3). Notably, in NSCLC, Lethio et al. identified six different subtypes of proteomes with distinct immune profiles, in addition to tumor mutational burden (TMB) and tumoral neoantigen burden (TNB), suggesting insights into the predictive potential of different types of checkpoint inhibitors [147]. Furthermore, by comparing genomic, transcriptomic, and proteomic data, valuable information can be obtained on the genetic diversity and evolutionary trajectories of tumors and metastases. This was highlighted in Ma et al.’s proteogenomic study on colorectal cancer (CRC), which used paired normal CRC, primary CRC, and liver metastatic tissues from samples collected from a clinical trial currently in the recruitment phase, which is associated with ID NCT02917707. This trial aimed to achieve a 5-year overall survival as the primary outcome and a 5-year disease-free survival as the secondary outcome. Ma et al. analyzed the data to identify specific signatures or protein mutations related to CRC and metastasis. By combining molecular alterations from WES and CRC cBioPortal, a customized protein database of CRC mutations was created to predict the prognostic potential of single amino acid variants (SAAVs) in CRC liver metastases [148]. As described in the following examples and summarized in Table 3, applications of proteogenomics in cancer research potentially allow the identification of tumor phenotypes, understanding of tumoral heterogeneity, and detection of patient-specific proteoforms as well as pathways and mechanisms responsible for cancer therapy success or resistance related to genomic and transcriptomic alterations [18].

1. High-Grade Serous Ovarian Cancer (HGSOC)

HGSOC is one of the most lethal gynecological cancers due to the inability to diagnose the disease at an early stage and frequent recurrences [154]. It is characterized by significant genomic and phenotypic heterogeneity. The substantial genomic instability and altered DNA repair mechanisms of HGSOC, known as “Homologus Repair Deficiency” (HRD), are related to different somatic and germinal mutations especially in “BReast Cancer gene 1/2” (BRCA1/2) genes [155]. This has led to the identification of specific drug therapies, such as poly (ADP-Ribose) polymerase inhibitors (PARPi), which exploit vulnerabilities in DNA repair pathways to induce tumor cell death. However, due to high molecular heterogeneity, 30% of HGSOC patients become resistant to therapy and relapse, often leading to death within five years of diagnosis [156,157].

In a recent study on the application of proteogenomics to HGSOC, Shrabanti Chowdhury et al. integrated genetic predictors, such as *BRCA1* inactivating mutations and loss of heterozygosity of chromosome 17, transcriptomic data, proteomic biomarkers and clinical features [149]. Genomic, transcriptomic, proteomic, and phosphoproteomic data were collected from three pre-treatment HGSOC tissue patient cohorts, which were divided into chemotherapy-refractory and sensitive to platinum/taxane therapy groups. Additionally, four public data repositories were used. The aim was to identify distinct proteogenomic signatures associated with chemo-refractory HGSOC.

By integrative analyses combining CNV, RNA, and global protein abundance data with multiple linear regression models, the relationships between molecular alterations in genes or proteins and platinum response were assessed.

Additionally, a predictive model of chemoresistance in HGSOC was developed using multiple machine learning models trained on proteins obtained from the analysis, previously deposited data, and literature. These models enabled the identification of sixty-four protein biomarkers related to chemo-refractory sensitivity, which could be useful for clinical therapeutic monitoring. Furthermore, starting from 150 pathways, five proteomic clusters were highlighted in both tissue biopsies and in vitro independent models [149]. Notably, a specific HGSOC subtype showed sensitivity to platinum-based therapy via pharmacological inhibition or CRISPR knockout of Carnitine Palmitoyl Transferase 1A (*CPT1A*), which is involved in a limiting step of fatty acid oxidation [149]. Therefore, for these subtypes, it would be more beneficial to use alternative therapeutic strategies such as metabolic inhibitors

2. Colorectal Cancer (CRC)

CRC is one of the most common and aggressive cancers affecting both in adult women and men [158]. Frequent recurrences and metastases result in a persistently low survival rate for patients due to high inter/intra-tumoral heterogeneity within CRC primary and metastatic tumor. This heterogeneity arises from the accumulation of genetic mutations, chromosomal aberrations, and environmental factors at the onset of disease and during its progression [159]. The genomic profiling of CRC reveals significant genomic instability characterized by CpG island methylator phenotype (CIMP), Chromosomal INstability (CIN) and Micro-Satellite Instability (MSI). CIN, CIMP and MSI forms are expressed differently based on tumor location—whether distal, rectal, or proximal—and these differences impact clinical therapy outcomes [158].

A recent proteogenomic study on both primary and metastatic CRCs collected from two cohorts of patients has significantly contributed to understanding metastatic progression [150]. They performed a discovery proteogenomic approach in the first cohort. Similarly, they conducted genomic, transcriptomic and proteomic integration in the second cohort, consisting of Fresh Frozen Tissue, including matched tumors at different stages with MSI and “Micro-Satellite Stable” (MSS) status, and normal tissues.

The study revealed six proteogenomic subtypes derived from three distinct subtypes in primary and metastatic CRC by using integrative unsupervised cluster analyses. These subtypes are characterized by hypoxia, stemness, and immune signatures. By suggesting specific mechanisms related to these pathways, this information may be useful for the clinical management of CRC and its progression [150].

Another recent proteogenomic study, involving deposited proteomic, genomic, and transcriptomic data from three CRC tissue and cell line databases, was conducted to characterize CRC linked to R-loop-Binding Proteins (RLBPs) [151]. Data on 204 RLBPs related to mRNA, CNA, or CpG promoter DNA methylation were analyzed using comprehensive statistical and cluster analyses, including non-negative matrix factorization. This analysis led to the identification of two distinct proteomic clusters with differential expressions of RLBPs [151].

The correlation analysis of these clusters with cell line drug sensitivity data revealed different RLBP profiles. One CRC cluster, characterized by the high expression of tumor-related RLBPs, showed greater sensitivity to therapeutic drugs targeting EGFR and genomic integrity compared to the second cluster, which exhibited low RLBP expression. Additionally, 42 differentially expressed RLBPs were identified across the CRC databases, highlighting their potential for further functional exploration in cancer progression and therapeutic applications.

3. Pancreatic Ductal Adenocarcinoma (PDAC).

PDAC is one of the most lethal and aggressive carcinomas, and it is often diagnosed at a locally advanced stage or after metastasis has occurred [160]. Surgical resection is currently the primary treatment modality for PDAC; however, only 15–20% of patients present with initially resectable tumors [161]. This low percentage is attributed to both the location and molecular heterogeneity of the tumor and its stroma, which also can complicate immunotherapy and chemotherapy treatments. The genomic profiling of PDAC shows an extreme genetic heterogeneity reflected in mutations across several genes [162]. A proteogenomic study on PDAC was conducted on a cohort of treatment-naive pancreatic tumor tissues, paired normal adjacent tissues, and normal pancreatic duct tissues from seven countries collected by the CPTAC program [152].

By combining genomic, transcriptomic, proteomic, and glycoproteomic data with statistical analyses, the researchers aimed to characterize PDACs and explore how genomic alterations impact transcript and protein abundances as well as PTMs. They identified differentially expressed proteins and glycoproteins in PDACs, which could serve as candidates for early detection.

In addition, another proteogenomic approach analyzed a cohort of 229 PDAC tumors along with paired non-tumor adjacent tissues. The clinical information for this cohort included age, TNM (tumor, nodes, metastasis) stage (I–III), various pathological conditions, survival rate (in months), and KRAS mutation status.

Proteogenomic characterization was performed by comparing WES, proteomic, and phosphoproteomic data with correlation analyses. This led to the identification of distinct proteomic and phosphoproteomic patterns related to genomic alterations. Specifically, the study revealed differential protein modifications related to *KRAS* mutations and the amplification of A Disintegrin and Metalloprotease 9 (*ADAM9*) [153]. Moreover, in an in vivo model, it was demonstrated that a higher frequency of *ADAM9* gene amplification could drive PDAC metastasis by reducing adhesion junctions and increasing WNT signaling pathway activity [153].

These studies, along with the others reported in Table 3, have shown proteogenomics as a powerful tool in detecting phenotypic features and their clinical impact on tumors.

#### Application of Single-Cell Multiomics Approaches on Cancer Studies

The advancement of single-cell DNA and RNA sequencing, Spatial Transcriptomic (ST) and the latest proteomic technologies has enabled the development of multiomics approaches at the single-cell level allowing for a more detailed description of intra-tumor heterogeneity.

Since 2013, when single-cell RNA sequencing was named “Method of the Year”, numerous studies have integrated this analysis, paving the way for new insights into molecular heterogeneity [163]. In 2019, a new “wave” of multimodal measurements emerged, extending beyond transcriptomic analysis, also to include the analysis of the methylome, chromatin modifications and surface proteins [164]. This trend continued with spatial transcriptomics, which was named “Method of the Year 2020” for its ability to retain the spatial information of individual cells, thus enhancing our understanding of the complex architectural heterogeneity of tissues [165]. However, most of these single-cell multimodal approaches combine two or three omics disciplines: integration of genomics and transcriptomics (mRNA–genome); transcriptomics and epigenomics (mRNA–chromatin accessibility or mRNA–DNA methylation) and transcriptomics with targeted proteomics (mRNA–protein data) [46,166].

For example, Bian et al. reported a single-cell triple omics sequencing approach in colorectal cancer cells where genomics, transcriptomics, and epigenomics data were simultaneously detected in single cells. Multidimensional scaling and unsupervised hierarchical clustering analyses were used to explore the integrated single-cell omics data. This integration allowed for the reconstruction of genetic lineages and traced the epigenomic and transcriptomic dynamics of primary and metastatic tumor cells [167].

This allowed for better awareness of the molecular alterations that occur during CRC progression and metastasis.

In 2022, Miheecheva et al. conducted an in-depth analysis of the TME subpopulations in clear cell renal cell carcinoma (ccRCC), incorporating genetic, proteomic, transcriptomic, and spatial information [168].

The study employed various technologies to obtain genomic, transcriptomic and proteomic data including CyTOF, Multiplex ImmunoFluorescence (MxIF), single-nucleus RNA sequencing (snRNA-seq), and bulk-level analyses with WES, RNA-seq, and methylation profiling. In addition, bioinformatic tools and machine learning algorithms were utilized to analyze and integrate these omic data. This approach revealed distinct CD4+, CD8+, and myeloid T-cell subpopulations as well as correlations between genetic alterations and TME composition.

Integrating multiomics data, such as transcriptomics and proteomics, in single-cell analyses could provide a clearer picture of the molecular alterations related to phenotypic heterogeneity. Advanced bioinformatic platforms and statistical analyses are essential for this purpose, as reported by Anjun Ma et al. in [169]. In this context, among these tools, Seurat and MOFA are widely used. The Seurat3 algorithms can integrate various types of data such as RNA expression with chromatin accessibility, other scRNA technologies, and cell-surface protein expressions. This integration enables the identification of cell-specific markers and provides a deeper understanding of the linkage between gene expression and protein abundance [169,170].

Conversely, Multi-Omics Factor Analysis (MOFA) is a computational tool designed to capture variations across multiple factors and multidimensional data. It helps to mitigate missing data and identify potential clinical markers and novel molecular drivers of heterogeneity [171].

Thus, these integrative multiomic approaches, combined with statistical and computational methods, help in correlating gene mutation and expression with protein levels, enhancing the understanding of tumor heterogeneity, and potentially leading to the development of more effective therapies, particularly in the field of immunotherapy.

For example, a study on NSCLC showed that the integration of scRNA-seq and CITE-seq analysis revealed a specific cellular module called the Lung Cancer Activation Module (LCAM), which was linked with TMB, tumor testis antigens, and *TP53* mutations [172].

The variability in LCAM levels among patients suggests that high LCAM may serve as a useful biomarker for predicting and monitoring responses to immune-modulating therapies.

Similarly, Bai et al. reported on the integration of scRNA-seq and CITE-seq analysis in CAR T-cells among pediatric patients with relapsed/refractory Acute Lymphoblastic Leukemia (ALL) [173]. The study revealed intrinsic phenotypic heterogeneity in CAR T-cell composition between long-term responders and relapsed ALL patients. This finding indicates that combining proteomic data with genomics and transcriptomics analyses could provide a comprehensive characterization of CAR T-cell populations, highlighting factors that may predict responses to CAR T-cell immunotherapy.

Another study by Gubin et al. integrated CyTOF and scRNA-seq for protein cellular and transcriptomic analyses, respectively [174]. Distinct cellular phenotypes across all hematopoietic cells of syngeneic mice tumors were identified during the administration of Immune Checkpoint Therapy (ICT). This integration provided new insights into the transcriptional, molecular, and functional changes that occur within lymphoid and myeloid immune cell populations, underscoring the importance of monitoring specific monocyte/macrophage populations after cancer immunotherapy [174].

Despite the growth in single-cell multiomics studies, many existing approaches rely on targeted methodologies, such as CyTOF and CITE-seq, where specific surface proteins or biomarkers are pre-selected. However, untargeted single-cell proteomics technologies are now emerging. Recent advances in techniques such as ScoPE-MS and ScoPE-MS2 have enabled the identification of a broader range of proteins and their regulatory interactions with transcripts, supporting hypothesis-free approaches [175,176]. These developments are paving the way for more comprehensive proteogenomic analyses at the single-cell level, enhancing our understanding of gene–mRNA–protein relationships across various tumor phenotypes.

## 3. Proteogenomics and Single-Cell Analyses: Criticisms and Challenges

Despite the significant advancements of proteogenomics in biomedical research, its workflow presents several challenges. As outlined in Figure 1 and discussed in Section 2, database construction is a critical component of the proteogenomics workflow [177].

Translating nucleotides into amino acid sequences for a customized protein database involves various methods, depending on the genomics or transcriptomics data used [33].

These methods can impact the size of the database and, consequently, the sensitivity of protein identification [178].

Protein identification by proteogenomics relies on an inference process, dependent on Peptide Spectrum Matches (PSMs) and the False Discovery Rate (FDR) threshold. Increasing the size of the customized database can lead to false positive identifications (PSM false) due to the higher number of potential spectral matches [178]. Conversely, using a stringent FDR threshold may result in false-negative PSMs, leading to an underestimation of protein abundance or loss of important information due to an incomplete database [178]. Therefore, different methodological strategies have been suggested to optimize analysis results [179,180,181]. These strategies include an “individualized” database that combines NGS and bottom–up proteomics analyses on the same samples. Furthermore, peptides validation is required after protein identification. This involves comparing identified peptides against major reference databases available for the organism of interest (e.g., RefSeq, UniProtKB, Ensembl) and common sample contaminants. To improve reproducibility and standardization, employing sophisticated dissection algorithms and automated software platforms is recommended [179,180,181]. Tools such as SpliceDB and CustomProDB for database creation, and ProGeo, Galaxy implementation PGtool, and Peppy for proteogenomics pipelines, have been developed to address these needs [182,183].

Despite the growing application of multiomics approaches, several technical, biological and computational limitations must be addressed in both single-cell analyses and LB analyses (Table 1 and Table 2).

Technical and biological variability pose significant challenges for both bulk and single-cell analyses Unlike bulk RNA-seq analyses, which have been extensively studied, scRNA-seq experiments are significantly impacted by technical factors such as RNA amplification bias and cell capture processes [52].

Single-cell proteomics face additional difficulties compared to genomics and transcriptomics because, unlike DNA and RNA, proteins cannot be amplified. Current single-cell proteomics techniques involve trade-offs between sensitivity throughput [48,53]. For instance, mass cytometry can measure up to 60 parameters simultaneously [54], and barcoding techniques like CITE-seq can process thousands of cells. However, these methods are constrained by the availability of high-quality antibodies. Immunoassay-based single-cell proteomic analyses, such as single-cell barcoding cytometry (SCBC) and single-cell Western blotting (scWB), are limited to detecting only a few proteins at a time.

In contrast, emerging SCP-MS techniques, such as SCoPE and SCoPE MS2, allow greater proteome coverage through multiplexing but still face challenges. Key issues include the isolation, digestion, and transfer of proteins to the mass spectrometer as well as maintaining high throughput without sacrificing coverage or incurring data loss [55,56].

Despite these challenges, ongoing improvements in sampling efficiency, ion accumulation and automation technologies [53] are opening up the possibility for more comprehensive and accurate single-cell proteogenomic analyses in the future [184].

To complement the discussion in Section 2.2.2, the following table summarizes the advantages, limitations, and troubleshooting strategies for various LB techniques.

## 4. Discussion and Conclusions

Tumor heterogeneity is a complex and pervasive characteristic that significantly influences tumor phenotypes.

Studies utilizing genomic and transcriptomic approaches—whether on solid tumor biopsies or through liquid biopsies—have illuminated the biological features of tumor evolution.

Moreover, NGS technologies applied at both tissue and single-cell levels have advanced our ability to characterize genetic and transcriptomic heterogeneity. These technologies enable the detailed exploration of the diverse molecular landscapes within tumors, providing insights into their evolution and behavior.

However, several mutations and transcriptomic alterations do not necessarily result in functional changes at a phenotypic (protein) level [18]. Therefore, a critical integration of data is essential for a comprehensive interpretation. In fact, protein heterogeneity is not solely a consequence of genetic or transcriptomic alterations. TME, immune system interactions, and the effects of specific drugs can modulate proteins expression and their modifications in ways that genomics or transcriptomics alone cannot always predict. Moreover, the complexity of protein heterogeneity can be further increased by PTMs and protein isoforms.

While NGS-based analyses such as those performed by the TCGA project have characterized most tumor types, they showed limitations in detecting all proteomic changes. Hence, to achieve a complete characterization of tumor heterogeneity, all phenotypic features such as proteins, protein networks, and PTMs has to be evaluated.

Indeed, recent advances in proteogenomics, driven by studies from CPTAC and improvements in computational methods for multiomic data integration, have moved beyond a sequencing-centric approach. These advancements have illuminated key cancer mechanisms [61,145,148,185].

For instance, in a study by Lethiö on NSCLC [147], while the tumor mutational burden was determined at the DNA level, proteomic data identified aberrant proteins caused by genomic aberrations in the tumor [186]. Therefore, proteogenomics can simultaneously enhance the understanding of cancer development pathways and immune evasion mechanisms [186].

The integration of multidimensional data, including molecular and clinical information, is facilitated by advanced statistical and bioinformatic tools, improving the biological understanding for patient stratification and precision treatments [187].

Proteogenomics reveals cancer signaling pathways and drug responses, and it identifies new therapeutic targets and biomarkers for diagnostic and therapeutic purposes [21,143,144].

For example, in an HGSOC study, proteogenomics revealed several subtypes sensitive to specific therapies, suggesting potential for diverse metabolic therapeutic approaches [149].

In CRC, proteogenomics enabled the identification of metastasis subtypes with different proteomic signatures [151], aiding early detection and targeted therapies [150]. In PDAC, known for its high molecular and phenotypic heterogeneity, proteogenomics identified key protein markers and mutations that may offer valuable biomarkers for targeted therapies and early detection [152].

Thus, proteogenomics represents a powerful approach for obtaining a comprehensive depiction of the molecular dynamics of intra-tumor heterogeneity with significant implications for diagnostic and therapeutic purposes.

Despite the promising advancements in proteogenomics, several challenges need to be addressed for clinical application. ITH remains a significant obstacle for molecular analyses in both clinical and preclinical models.

Additionally, constructing comprehensive databases, achieving accurate protein identification, and the lack of standardized protocols continue to challenge the proteogenomic workflow.

Nevertheless, organizations like TCGA, CPTAC, and the International Cancer Proteogenomic Consortium (ICPC) are actively collaborating to establish standardized proteogenomic pipelines to enhance this approach for clinical use [185,188].

For instance, CPTAC has employed phosphoproteomics and targeted MS approaches such as Multiple Reaction Monitoring [18,141]. Moreover, advancements in bioinformatics tools and platforms, including CustomProDB, NetGestalt, LinkedOmic and iProFun, have made integrative proteogenomic data analyses and sharing more accessible [142,145]. Finally, proteogenomic workflows are also advancing the context of organoids and single cells analyses, increasing the potential of proteogenomics in clinical and biomedical research [51,123,125,132,133]. The advancement of single-cell multiomics has significantly deepened the understanding of tumor heterogeneity.

Although single-cell proteomic analyses have rapidly evolved, they are still in the early stages, revealing only the “tip of the iceberg” [189].

Addressing both technical and biological challenges would be useful for further applications of single-cell proteogenomic approaches to better decipher molecular and phenotypic changes in cancer cells.

In conclusion, despite its current limitations, proteogenomics remains a fundamental and innovative approach for tumor phenotyping. By identifying specific drivers mutation, new genomic regions, protein signatures (such as neoepitopes and proteoforms), and potential immuno-therapeutic targets, proteogenomics lays the groundwork for a more profound characterization of tumor phenotypes and paves the way for more personalized medicine strategies.

## Figures and Tables

**Table 2:** Summary of advantages, limitations and troubleshooting of LB techniques.

Techniques	Advantages	Limitations and Troubleshooting	References
ddPCR	High sensitivity and specificity for target mutation; low cost/reaction; no replicate reactions and standard curve	High cost for instrumental implementation; single- or low-plex analysis; need for PCR optimization strategies; need for positive/negative controls	[83,84,85]
BEAMing	High sensitivity and specificity for target mutation; low cost/reaction	Single- or low-plex analysis; potential background noise and false positive risk; need of bead based PCR optimization strategies; need for stringent controls	[83,84,85]
CAPP-Seq	High sensitivity; higher multiplexing analysis; detection of all the major mutation types; hybrid capture-based method not dependent on fragment size; cost-effective	Potential inefficient capture of fusions; potential false positive; need for large input	[83,86]
WGBS-Seq	Gold standard for methylome analysis	Expensive for large sample number; reduced sensitivity due to DNA degradation; higher read depth needed to improve sensitivity	[83]
TAm-Seq	High specificity and sensitivity; higher multiplexing analysis; reduced time and cost	Amplicon-based methods depend on fragment size; less sensitive compared to individual loci assays; higher read depth needed to improve sensitivity	[83,87]
WES	Comprehensive analysis of coding region; low cost; high yield	Low sensitivity; need of advanced bioinformatics tools to manage and analyze output datasets; higher read depth needed to improve sensitivity	[83,84]
WGS	Comprehensive analysis of tumor mutations types; potential for detailed mutational landscape	Time and cost consuming; variable sensitivity and specificity; need of stringent quality assurance; ethical issues; need of advanced bioinformatics tools to manage and analyze output datasets	[83,84]
MS	Non-invasive and sensitive; complete characterization of proteins and PTMs, over a thousand proteins in blood, thousands in urine	High dynamic range of blood protein content; pre-analytical variations; require depletion protocols; using robust MS approaches (DIA)	[57,88]
RPPA	Robust in parallel large sample profiling, sensitive, cost effective	Requires high-quality antibodies, complex experimental workflow, prolonged process; validation of antibodies, optimize signals and workflow	[27,88,89]
SOMAscan	High-affinity protein-binding reagents, expands targeted proteomics toolkit	Limited aptamers compared to antibodies, preliminary exploration of PTM biomarkers; developing more high-quality aptamers, advancing PTM-oriented aptamer development	[28,88]

ddPCR: droplet digital polymerase chain reaction; BEAMing: Beads Emulsion Amplification and Magnetics; TAm-Seq: Tagged-Amplicon deep Sequencing; CAPP-Seq: CAncer Personalized Profiling by deep Sequencing; WGBS-Seq: Whole Genome Bisulfite Sequencing; WES: Whole Exome Sequencing; WGS: Whole Genome Sequencing; MS: mass spectrometry; DIA: Data-Independent Acquisition; RPPA: Reverse Phase Protein Array; SOMAscan: Slow Off-rate Modified Aptamer scan.

**Table 3:** Summary of several proteogenomics approaches on cancer. This table provides an overview of several proteogenomic approaches used to investigate tumor heterogeneity in different cancer types, including colorectal cancer (CRC), high-grade serous ovarian cancer (HGSOC), non-small cell lung cancer (NSCLC), lung adenocarcinoma (LUAD) and pancreatic ductal adenocarcinoma (PDAC).

Model Study	Proteogenomic Techniques	Applications	Aims	References
tissue biopsies (OCT)	(WGS), (RNA-Seq) and global proteomic and phosphoproteomic analyses (TMT LC-MS/MS) and RRPA.	LUAD	(-) to identify protein and RNA signatures predicting survival of patients. (-) to identify potential therapeutic vulnerabilities (proteogenomic signatures) among subtypes by proteomics and phosphoproteomics networks.	[146]
tissue biopsies (FFT)	Panel sequencing, DNA methylation analysis, proteomic analyses (TMT LC-MS/MS and DDA/DIA LC-MS/MS) and synthetic peptide analysis.	NSCLC	(-) to detect molecular phenotypes and cancer-related proteins for the identification of specific cancer dependencies and immune-evasion mechanisms.	[147]
tissue biopsies (FFT)	(WES), (RNA-Seq), single nucleotide polymorphism array and proteomic analyses (LC-MS/MS).	CRC	(-) to characterize molecular heterogeneity of colorectal cancer and liver metastasis. (-) to predict functional correlation with genomic abnormalities for potential prognostic value.	[148]
tissue biopsies (FFT and FFPE) and PDX	(WGS), (RNA-Seq), MSK-IMPACT data, proteomic, phosphoproteomic and targeted proteomic analyses (TMT LC-MS/MS and MRM-MS).	HGSOC	(-) to identify distinct proteogenomic signatures that predicts chemotherapy-refractory cancers and implicates potential therapeutic vulnerabilities.	[149]
tissue biopsies (FFT and FFPE)	(WGS), (RNA-Seq), MSK-IMPACT targeted cancer gene sequencing and proteomic analysis (LC-MS/MS).	CRC	(-) to identify distinct proteogenomic subtypes of colorectal cancer characterize primaries and liver metastases. (-) to study tumor progression and its heterogeneity.	[150]
three colorectal cancer databases, cell lines databases and PDO	Single-cell data, DNA methylation, RNA, and copy number alteration data along with global TMT LC-MS/MS proteomic data.	CRC	(-) to identify significant prognostic biomarkers and potential therapeutic targets. (-) to characterize different CRC subtypes associated with R-loop binding proteins.	[151]
tissue biopsies (FFT)	(WES), (WGS), (RNA-Seq), DNA methylation analyses, miRNA sequencing, proteomic, phosphoproteomic and glycoproteomic analyses (TMT LC-MS/MS and DIA LC-MS/MS).	PDAC	(-) to delineate phenotypic effects related to genomics and epigenomics aberrations in PDAC for identification of potential novel therapeutic targets. (-) to detect proteogenomic features, clinical biomarker of PDAC subtypes and specific to neoplastic ductal epithelial cells.	[152]
tissue biopsies (FFPE), 2D in vitro model	(WES), (RNA-Seq), proteomic and phosphoproteomic analyses (LC-MS/MS).	PDAC	(-) to decipher the impact of genomic alterations in gene expression, protein abundance, and phosphorylation modification for prognostic value. (-) to monitor PDAC cancer development and progression by in vivo functional experiments.	[153]

FFT: Fresh Frozen Tissue; FFPE: Formalin-Fixed Paraffin-Embedded; OCT: Optimal Cutting Tissue; PDO: Patient-Derived Organoid; PDX: Patient-Derived Xenograft; 2D in vitro model: Two-Dimensional cell-cultures model; WGS: Whole Genome Sequencing; WES: Whole Exome Sequencing; RNA-seq: RNA sequencing; MSK-IMPACT: Memorial Sloan Kettering-Integrated Mutation Profiling of Actionable Cancer Targets; miRNA: microRNA; TMT: Tandem Mass Tag; LC-MS/MS: Liquid Chromatography Mass Spectrometry; DDA: Data-Dependent Acquisition; DIA: Data-Independent Acquisition; MRM: Multiple Reaction Monitoring; MS: Mass Spectrometry.

## Data Availability

No new data were created or analyzed in this study.
